# Transient Receptor Potential Vanilloid 4 Channel Deficiency Aggravates Tubular Damage after Acute Renal Ischaemia Reperfusion

**DOI:** 10.1038/s41598-018-23165-0

**Published:** 2018-03-20

**Authors:** Marwan Mannaa, Lajos Markó, András Balogh, Emilia Vigolo, Gabriele N’diaye, Mario Kaßmann, Laura Michalick, Ulrike Weichelt, Kai M. Schmidt–Ott, Wolfgang B. Liedtke, Yu Huang, Dominik N. Müller, Wolfgang M. Kuebler, Maik Gollasch

**Affiliations:** 1 Charité Campus Virchow, Nephrology/Intensive Care, Berlin, Germany; 2 Experimental and Clinical Research Center, a joint cooperation between the Charité Medical Faculty and the Max–Delbrück Center for Molecular Medicine, Berlin, Germany; 3 Department of Medical Biology, University of Pécs Medical School, Pécs, Hungary; 4 Signal Transduction Research Group, János Szentágothai Research Centre, Pécs, H-7624 Hungary; 5 Max–Delbrück Center for Molecular Medicine, Berlin, Germany; 6 Institute of Physiology, Charité Universitätsmedizin Berlin, Berlin, Germany; 7 Departments of Neurology, Neurobiology, and Clinics for Pain and Palliative Care, Duke University Medical Center, Durham, USA; 8 Institute of Vascular Medicine, Chinese University of Hong Kong, Hong Kong, China; 9 Li Ka Shing Institute of Health Sciences, Chinese University of Hong Kong, Hong Kong, China

## Abstract

Transient receptor potential vanilloid 4 (TRPV4) cation channels are functional in all renal vascular segments and mediate endothelium-dependent vasorelaxation. Moreover, they are expressed in distinct parts of the tubular system and activated by cell swelling. Ischaemia/reperfusion injury (IRI) is characterized by tubular injury and endothelial dysfunction. Therefore, we hypothesised a putative organ protective role of TRPV4 in acute renal IRI. IRI was induced in TRPV4 deficient (*Trpv4* KO) and wild–type (WT) control mice by clipping the left renal pedicle after right–sided nephrectomy. Serum creatinine level was higher in *Trpv4* KO mice 6 and 24 hours after ischaemia compared to WT mice. Detailed histological analysis revealed that IRI caused aggravated renal tubular damage in *Trpv4* KO mice, especially in the renal cortex. Immunohistological and functional assessment confirmed TRPV4 expression in proximal tubular cells. Furthermore, the tubular damage could be attributed to enhanced necrosis rather than apoptosis. Surprisingly, the percentage of infiltrating granulocytes and macrophages were comparable in IRI–damaged kidneys of *Trpv4* KO and WT mice. The present results suggest a renoprotective role of TRPV4 during acute renal IRI. Further studies using cell–specific TRPV4 deficient mice are needed to clarify cellular mechanisms of TRPV4 in IRI.

## Introduction

Transient receptor potential (TRP) channels were initially identified in a mutant strain of *Drosophila melanogaster* as a novel component required in phototransduction^1^. Subsequently, they were identified in vertebrates and found to be ubiquitously expressed in many cells and tissues. Currently the mammalian TRP channel family comprises 28 members which share some structural similarity with each other^2^. Mutated TRP genes are causal for human hereditary diseases, the so–called ‘TRP channelopathies’^3^, for some TRP genes.

TRP vanilloid 4 (TRPV4) [initially named vanilloid receptor–related osmotically activated ion channel (VROAC), vanilloid receptor–like channel 2 (VRL–2), TRP protein 12 (TRP12), and osmosensory protein 9–like transient receptor potential channel, member 4 (OTRPC4)] represents the fourth member of the TRPV subfamily, and was originally identified as a mammalian homolog of the *Caenorhabditis elegans* OSM–9^3–6^. TRPV4 is a polymodal, non–selective cation channel, which was initially demonstrated to be activated by hypotonicity–induced cell swelling^7,8^. Later studies revealed that it can also be activated by various other stimuli including mechanical stimulation^6,9^, moderate heat, endogenous chemicals such as anandamide, arachidonic acid and its epoxyeicosatrienoic acid metabolites, as well as by a number of exogenous chemical ligands^10–12^ or UVB^13,14^. Human *TRPV4* mutations are known to cause skeletal dystrophic disorders of varying severity, and neurological conditions, spinal muscular atrophies and the predominantly motor-neuropathy, Charcot-Marie Tooth Disease, type 2C^12^.

Expression of TRPV4 in mammals is wide-spread. It is expressed in the central and peripheral nervous system^15^, noticeably in tissues involved in volume regulation and osmosensing such as the circumventricular organs, the vascular endothelium and the kidney^12,16,17^. In an earlier study expression of TRPV4 in the kidney has been reported to be restricted to the distal part of the renal nephron system, which is constitutively or conditionally water–impermeable^18^, thereby supporting its osmoregulatory role^17^. In other studies, TRPV4 channel has been shown to be activated by hypotonic cell swelling, which further suggested that TRPV4 functions in response to osmotic cues^19^. Indeed, osmotic and mechanical sensing are impaired in *Trpv4*-/- mice^8,17^. A later study showed that TRPV4 is also expressed in renal connecting tubule/collecting duct cells and is activated by fluid flow^20^. Flow-induced activation of TRPV4 in the thick ascending limb indicates that TRPV4 channels might be involved in salt homoeostasis^21^. Pharmacological and siRNA experiments suggested that hypotonicity-induced TRPV4 activation is a prerequisite for ATP release from epithelial cells in response to osmotic changes^19^. More recent data demonstrate a functional interaction between TRPV4 and aquaporin 2 in response to anisosmotic conditions^22^.

On the other hand, TRPV4 channels in endothelial and smooth muscle cells have been recognized to play an important role in the control of vascular tone^23,24^. Recently, we have shown that TRPV4 promotes endothelium-dependent relaxation in renal resistance arteries, renal conduit arteries and in medullary vasa recta^25^. TRPV4 has been shown to function as key endothelial ion channels for calcium influx to control vascular function^26^. Moreover, TRPV4 participates in hypoxic vascular relaxation which is critical for postischaemic ‘no–reflow’ tissue regions^27^. The outcome of acute kidney injury (AKI) has been primarily linked to renal tubular injury. In fact, most theories consider renal tubular cells as the main culprit, i.e, tubular obstruction, calcium overload, loss of cytoskeletal integrity, and loss of cell–matrix adhesion due to AKI^28–30^. However, more than forty years ago Flores and colleagues pointed out that ischaemic swelling of endothelial cells may occlude the flow in small blood vessels, which prevents reflow even if the initial cause of ischaemia is relieved. This hypothesis was supported by several other studies^31–33^ and these studies established the pathophysiological role of endothelial cell activation and dysfunction in AKI.

TRPV4 channels present a promising novel target to treat acute and chronic renal disease processes^34^. In line with the above described vasodilatory properties and the regional and functional distribution of TRPV4 in the renal vasculature and tubular system, we hypothesized that TRPV4 might function in a renoprotective manner in renal ischaemia/reperfusion injury (IRI). To elucidate the putative role of TRPV4 in AKI, we performed renal IRI studies using *Trpv4* KO mice^17^ and wild–type (WT) control mice as littermates on the same genetic background. We show that *Trpv4* deficiency deteriorates the immediate outcome of AKI, in that renal – and primarily cortical – tubular damage is aggravated in *Trpv4* deficient mice after IRI which was attributable to increased necrosis rather than apoptosis.

## Results

### The Impact of TRPV4 Deficiency on Renal Damage after IRI

**Figure 1:**
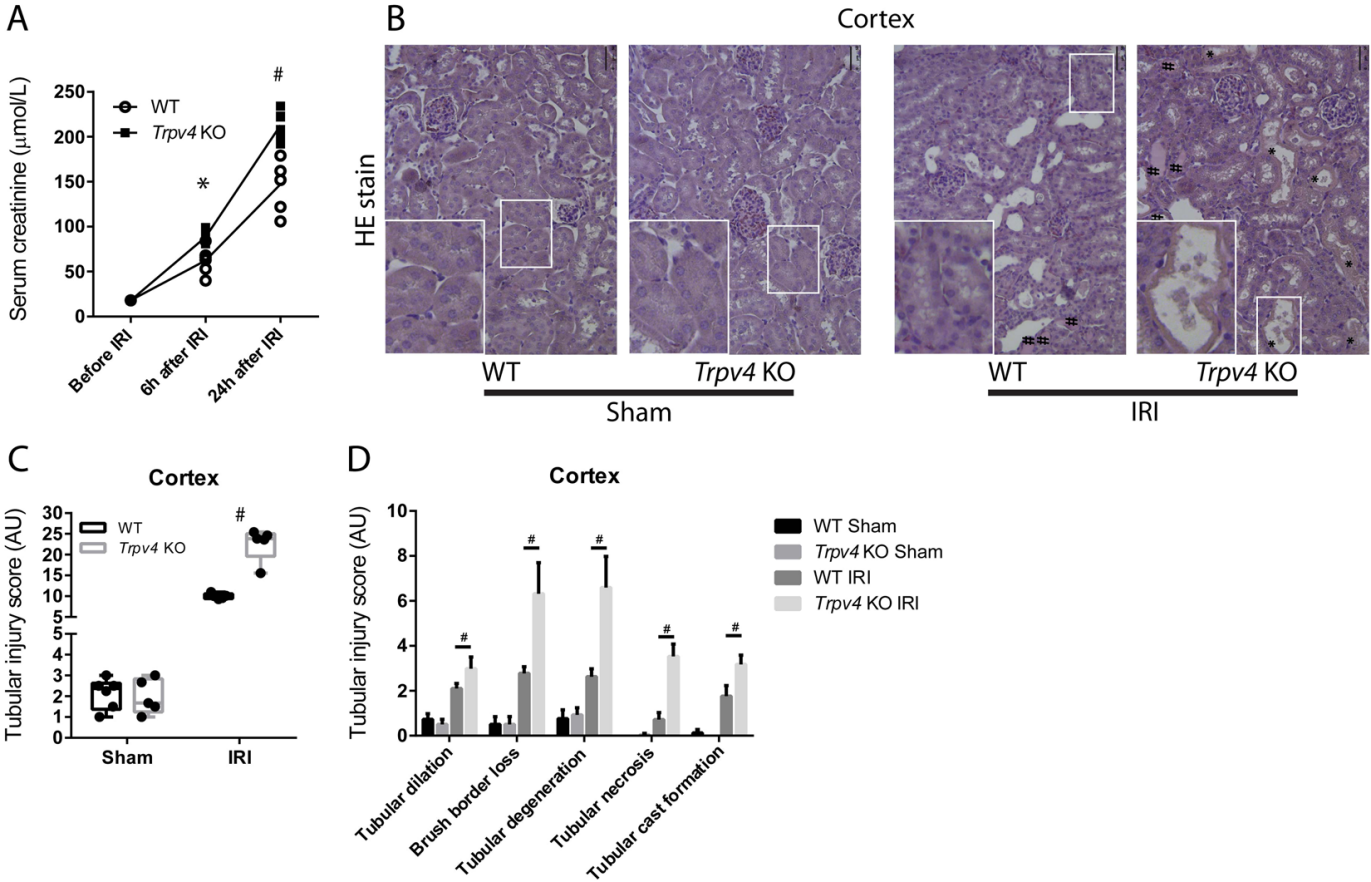
Renal function and damage after renal ischaemia/reperfusion injury (IRI). (**A**) Serum creatinine levels of *Trpv4* KO (▪) and WT (○) mice at baseline, 6 and 24 hours after renal IRI. Please note that at baseline all serum creatinine levels were below the measurement limit (18 µmol/L). n = 6 for both WT and *Trpv4* KO mice, repeated measurement two-way ANOVA (P_Time_ < 0.0001, P_Genotype_ = 0.0007, P_Interaction_ < 0.0001), Sidak’s multiple comparisons test, *P < 0.05, ^#^P < 0.001. (**B**) Representative cortical images of haematoxylin and eosin stained sections of sham and I/R–injured kidneys of WT and *Trpv4* KO mice (×200). * on the histological images represent necrosis and ^#^ represent tubular cast formation. (**C**) Semi–quantification of cortical tubular injury in sham and I/R–injured kidneys of WT and *Trpv4* KO mice, two-way ANOVA (P_Treatment_ < 0.0001, P_Genotype_ = <0.0001, P_Interaction_ < 0.0001), Sidak’s multiple comparisons test, ^#^P < 0.001, n = 6 for both WT and *Trpv4* KO mice. (**D**) Detailed analysis of cortical tubular damage, one-way ANOVA, Tukey post-hoc test, ^#^P < 0.001, n = 6 for both WT and *Trpv4* KO mice.

To gain insights into the (patho)physiological role of TRPV4 in renal IRI, we performed comparative *in vivo* studies using TRPV4 deficient (T*rpv4* KO) mice and wild–type (WT) control mice. Although *Trpv4* KO mice exhibited slightly higher serum concentrations of sodium and glucose, and blood haemoglobin and haematocrit levels at baseline, the kidney function markers creatinine and urea nitrogen were initially indistinguishable between *Trpv4* KO and WT mice (Table 1). Already six hours after renal IRI, however, *Trpv4* KO mice showed higher serum creatinine and urea nitrogen levels compared to WT mice; this difference in creatinine levels persisted over a period of twenty–four hours after ischaemia (Fig. 1A). Other lab parameters summarised in Supplementary Table 1 showed no significant differences at these time points after renal ischaemia. Sham–operated *Trpv4* KO mice had similar creatinine levels compared to sham-operated WT mice (Supplemental Fig. 1). Kidney sections were haematoxylin and eosin (HE) stained to assess the degree of tubular damage after ischaemic AKI. Histological analyses and semi–quantitative scoring revealed aggravated cortical tubular damage in IRI–damaged kidneys of *Trpv4* KO mice compared to IRI–damaged kidneys of WT mice (Fig. 1B,C). Of the five examined parameters tubular necrosis, tubular degeneration and brush border loss showed the most prominent differences between IRI–damaged kidneys of WT and *Trpv4* KO mice, whereas sham-operated mice did not differ in any of the examined parameters (Fig. 1D). Extensive necrosis of tubular cells with brush border was verified by proximal tubular cell specific staining for aquaporin-1 (Supplemental Fig. 2). Additionally, we verified the existence of functional TRPV4 channels in the proximal tubular system. Proximal tubular cells (PTCs) were isolated from WT and *Trpv4* KO mice and were stimulated *in vitro* with the TRPV4 agonist GSK1016790A at both 10 and 100 nM concentrations after loading with the calcium indicator fluo-4 AM. Calcium influx was detected in PTCs of WT mice after application of GSK1016790A but not in PTCs of *Trpv4* KO mice (Supplemental Fig. 3). Histological analysis of the outer stripe of the outer medulla, a region characterized by a relative paucity of intertubular capillaries and therefore particularly prone to ischemic injury, showed less distinctive, but similar differences (Supplemental Fig. 3A–C).

**Table 1:** Baseline serum parameters of WT and *Trpv4* KO control mice. n = 6 n = 4 for WT and n = 5 for Trpv4 KO mice, two-tailed unpaired t-test. n.a. = not applicable.

Serum parameter	Unit	WT		*Trpv4* KO		P-value
		Mean	SD	Mean	SD	
Sodium	mmol/L	145,86	1,07	148,00	1,41	0,019
Potassium	mmol/L	4,71	0,42	5,05	0,82	0,382
Chloride	mmol/L	113,00	2,58	115,50	1,29	0,108
Ionized Calcium	mmol/L	1,28	0,05	1,28	0,04	1,000
Total Carbon Dioxide	mmol/L	21,57	0,98	18,00	2,45	0,007
Glucose	mg/dL	203,71	19,31	236,50	18,45	0,023
Urea Nitrogen	mg/dL	7,49	1,89	9,25	0,54	0,108
Creatinine	umol/L	<18	n.a.	<18	n.a.	n.a.
Hematocrit	% PCV	38,71	2,06	41,50	0,58	0,029
Hemoglobin	g/dL	13,15	0,73	14,10	0,23	0,035
Anion Gap	mmol/L	16,71	1,98	20,25	1,26	0,011

**Figure 2:**
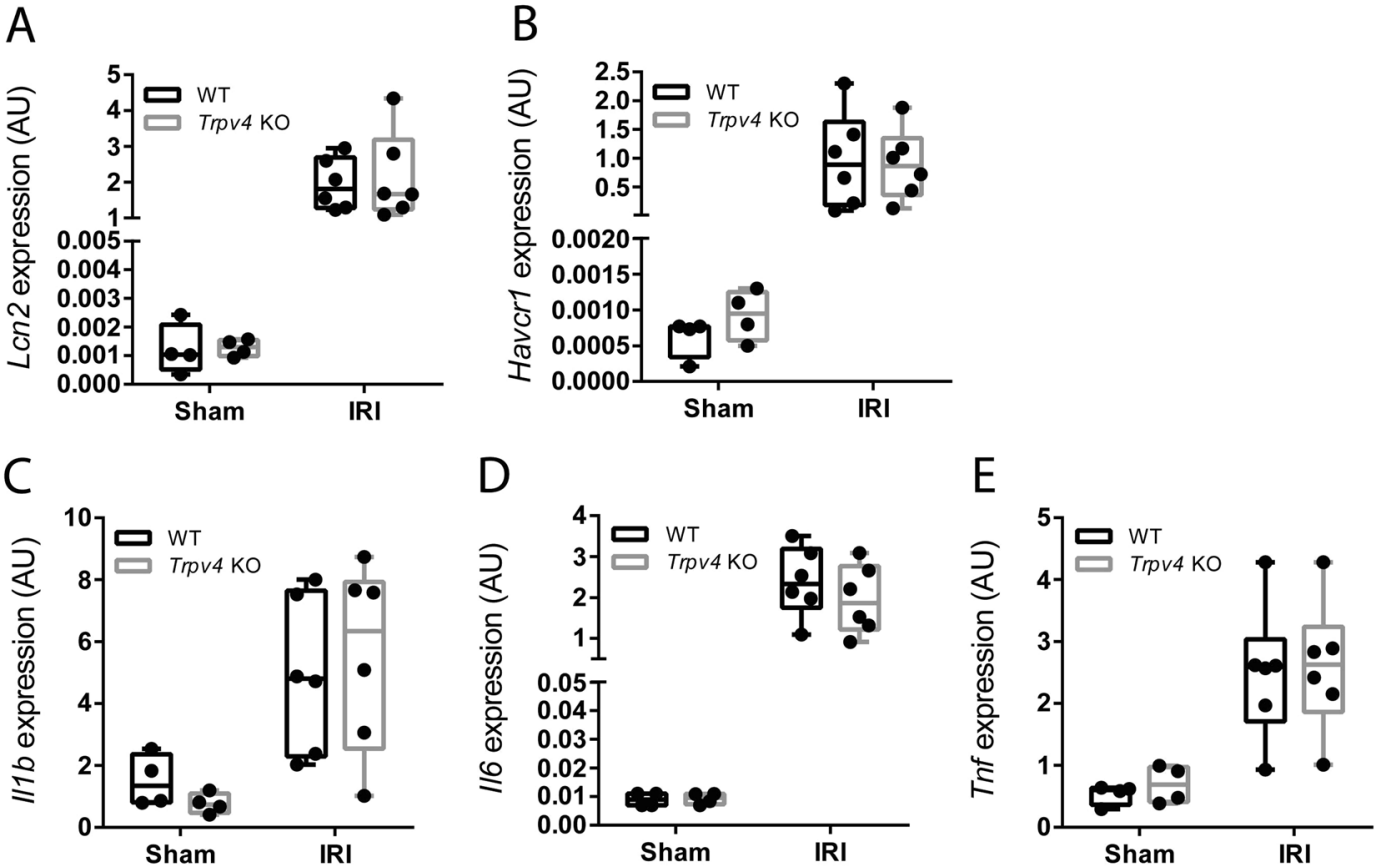
Expression of renal injury and pro-inflammatory markers. (**A**) Renal mRNA levels of lipocalin 2 (*Lcn2*; also known as neutrophil gelatinase–associated lipocalin [*Ngal*]), (**B**) kidney injury molecule 1 (*Kim1*) and pro–inflammatory cytokine (**C**) interleukin 1 beta (*Il1b*) and (**D**) *Il6* and (**E**) tumor necrosis factor (*Tnf*) in sham and in *Trpv4* KO and WT mice following IRI. Renal gene expression data are determined in n = 4 for sham-operated WT and *Trpv4* KO mice and n = 6 for I/R–injured WT and *Trpv4* KO mice, two-way ANOVA. In all cases P_Treatment_ < 0.001, and P_Genotype_ and P_Interaction_ are not significant.

Surprisingly, renal mRNA levels of the AKI marker lipocalin 2 (*Lcn2*; also known as neutrophil gelatinase–associated lipocalin [*Ngal*]) and kidney injury molecule-1 (*Kim1*, also named *Tim1* and *Havcr1*), as well as renal mRNA levels of the pro–inflammatory cytokines interleukin (*Il*)–1 beta, *Il*–6 and tumor necrosis factor (*Tnf*) did not differ between sham or IR-injured kidneys of *Trpv4* KO and WT mice (Fig. 2A–E).

### Expression of TRPV4 in the Renal Tubular System

**Figure 3:**
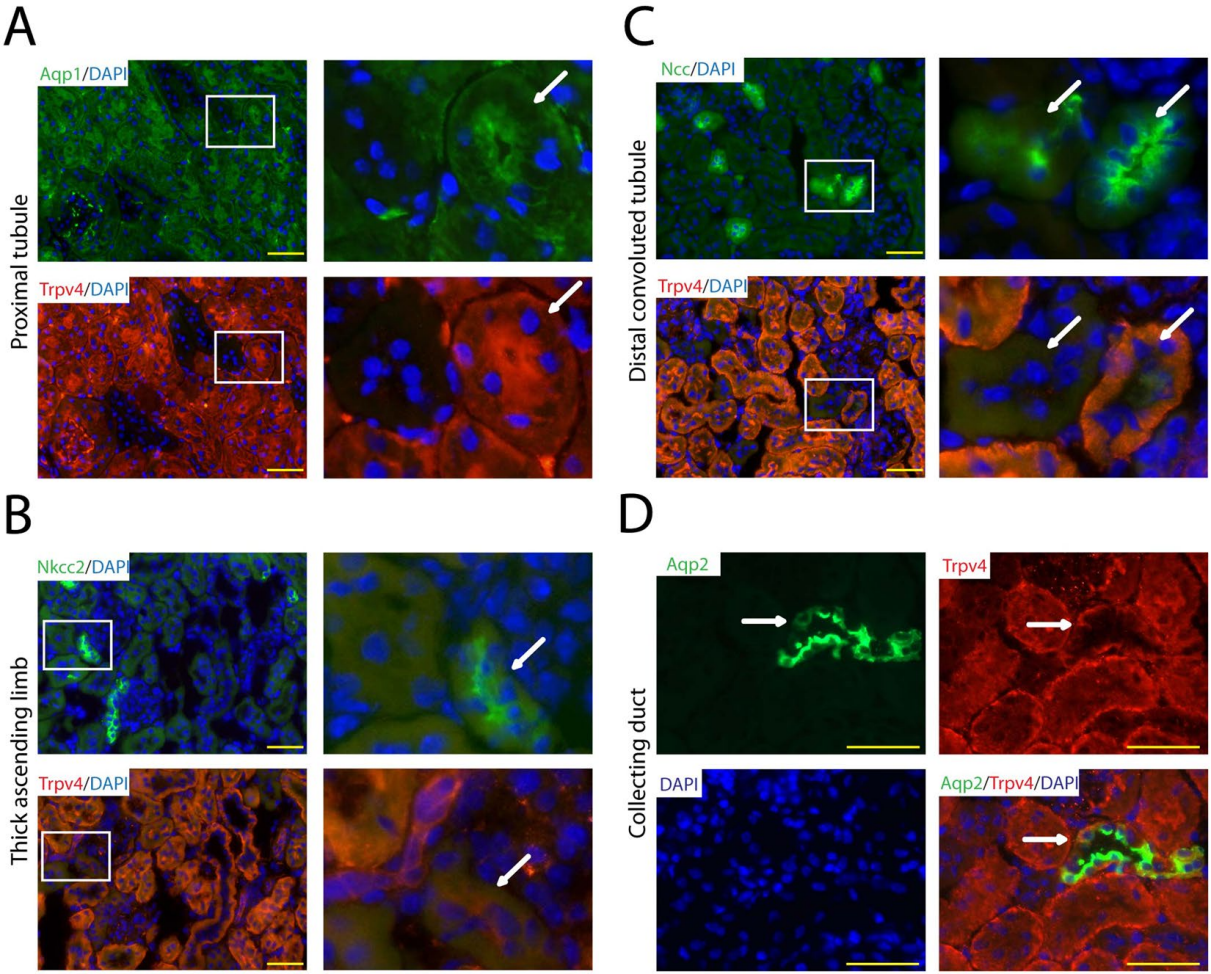
Analysis of renal tubular TRPV4 expression in the kidney of WT mice by immunostaining. (**A**) The proximal tubule marker aquaporin-1 (Aqp1), (**D**) the collecting duct marker aquaporin-2 (Aqp2) and (**C**) partly the distal convoluted tubule marker sodium-chloride symporter (Slc12a3) co-localize with TRPV4. (**B**) The thick ascending limb marker sodium-potassium-chloride cotransporter 2 (Slc12a2) does not co-localize with TRPV4. White squares mark equivalent tubular sections represented on adjacent sections. White arrows point to the same tubular structures seen on two serial cuts stained by different antibodies. Yellow bar represents 50 μm.

We were somewhat surprised of the massive tubular necrosis and brush border loss seen in the renal cortex of *Trpv4* KO mice, as it is believed that TRPV4 is not expressed in proximal tubular cells. Therefore, we used the advantage of our KO mice and tested several commercially available antibodies against TRPV4, however, only one of the tested commercially available antibodies worked in our hands for immunofluorescence detection of TRPV4 (Supplemental Fig. 4). A detailed analysis of renal tubular TRPV4 expression in WT kidneys revealed that TRPV4 is co-expressed in aquaporin-1 (proximal tubule), aquaporin-2 (collecting duct) and partly in sodium-chloride symporter (distal convoluted tubule) positive tubules but not in sodium-potassium-chloride co-transporter 2 (thick ascending limb) positive tubules (Fig. 3A–D).

### The Impact of TRPV4 Deficiency on Renal Apoptosis after IRI

**Figure 4:**
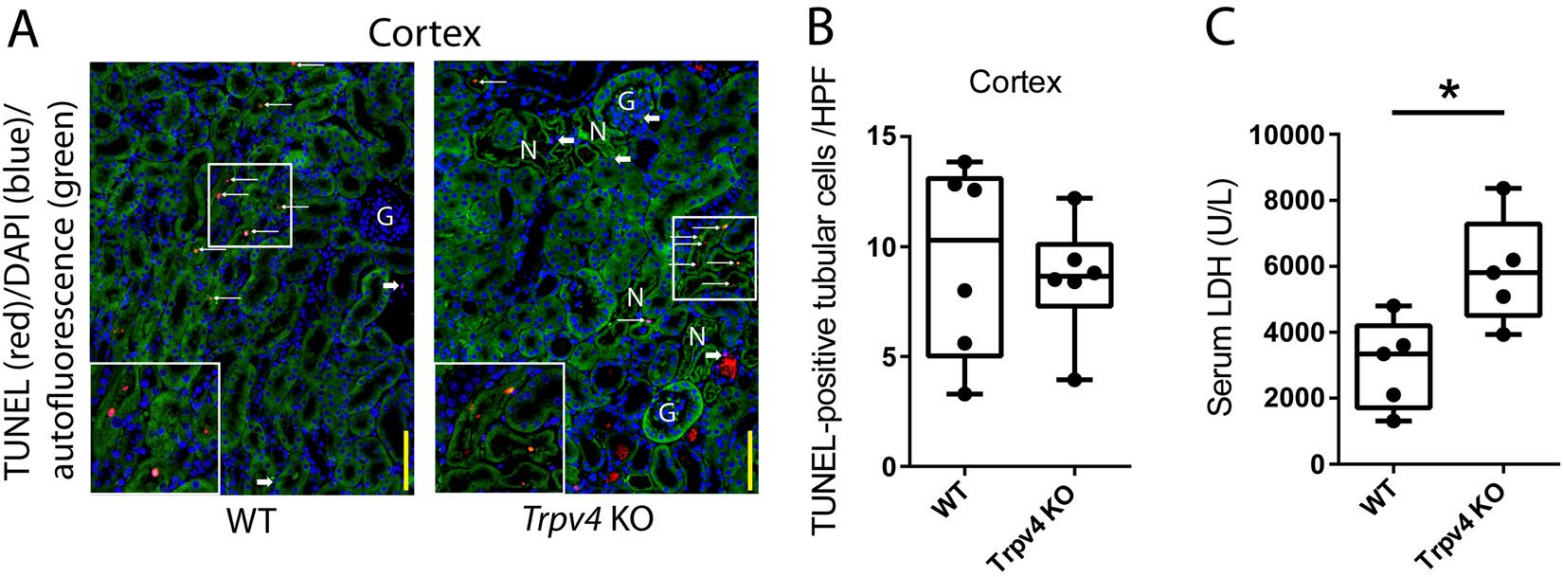
TRPV4 deficiency does not alter cortical tubular apoptosis upon acute ischaemic kidney injury. (**A**) Representative ×200 images of TUNEL labelling on WT and *Trpv4* KO kidney sections 24 hours after renal ischaemia. Thin white arrows indicate TUNEL–positive tubular cells. Thick white arrows indicate TUNEL–positive interstitial cells. Yellow bars represent 100 μm. (**B**) Number of TUNEL–positive cortical tubular cells per ×200 field. Each dot represents a single animal and is the mean of 5 randomly selected cortical fields. n = 6 for both WT and *Trpv4* KO mice, two-tailed unpaired t-test. G, glomerulus; N, necrosis. (**C**) Serum lactate dehydrogenase (LDH) levels in WT and *Trpv4* KO mice 24 hours after renal ischaemia. n = 5 for both WT and *Trpv4* KO mice, two-tailed unpaired t-test, *P < 0.05.

In renal ischaemia, there is mismatch of local tissue oxygen supply and demand and cells accumulate metabolic waste products. As a result of this imbalance, tubular epithelial cells undergo death by apoptosis or necrosis depending on injury severity. Moreover, renal tubular epithelial apoptosis can initiate reperfusion–induced inflammation and subsequent tissue injury^35^. To assess tubular epithelial apoptosis, TUNEL labeling was performed on paraffin sections of IRI–kidneys of WT and *Trpv4* KO mice. Interestingly, injured kidneys of *Trpv4* KO mice showed similar rates of apoptosis as injured kidneys of WT mice (Fig. 4A). Quantification by direct counting of TUNEL–positive tubular cells confirmed this observation (Fig. 4B). Nevertheless, the concentration of the tissue damage marker lactate dehydrogenase (LDH) was significantly higher in IRI-injured *Trpv4* KO mice compared to WT mice, supporting the histological finding of more extensive renal necrosis in *Trpv4* KO mice (Fig. 4C). Contralateral kidneys of *Trpv4* KO and WT mice showed virtually no TUNEL–positive tubuli (Supplemental Fig. 5).

### The Impact of TRPV4 Deficiency on Renal Cellular Infiltration after IRI

**Figure 5:**
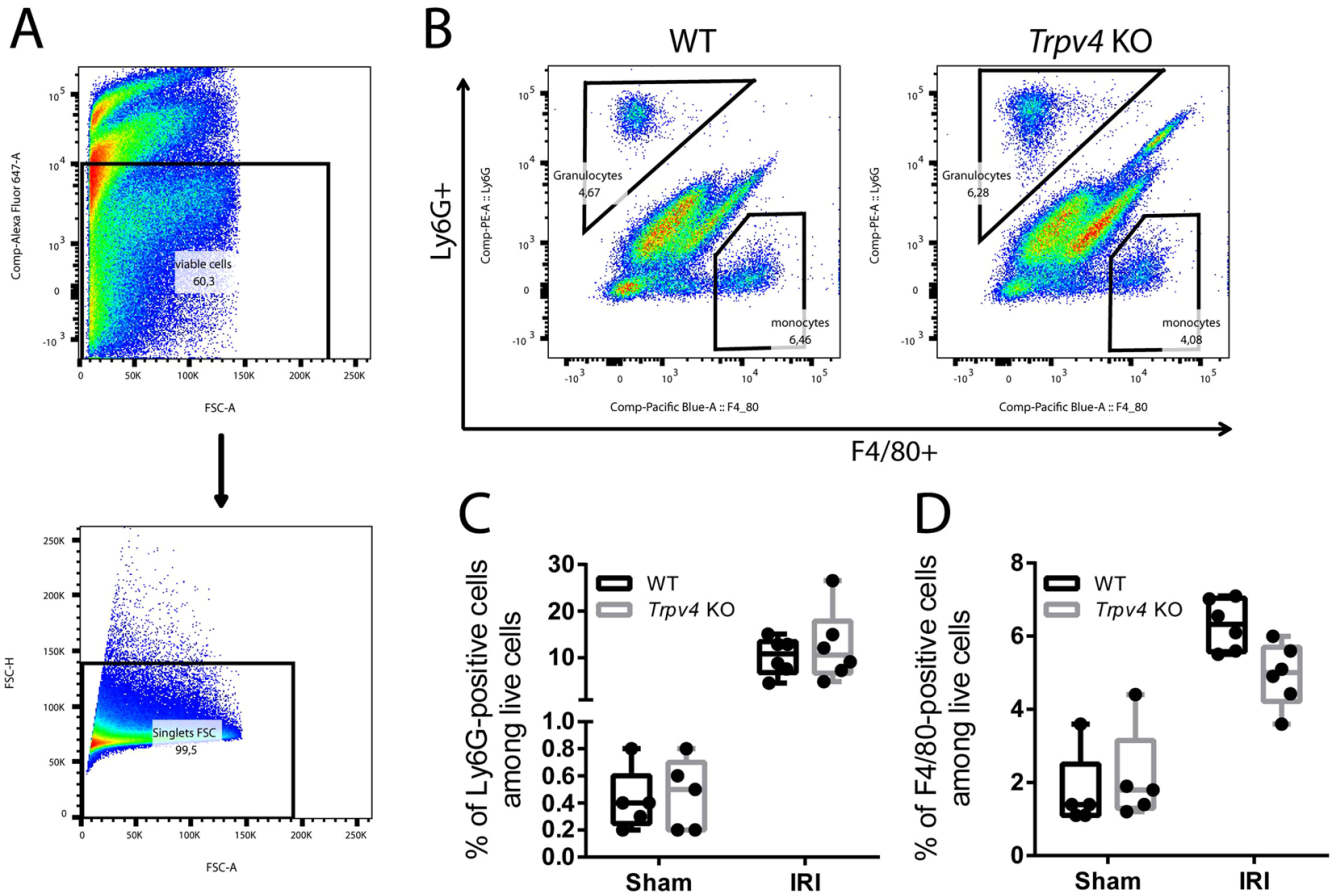
Flow cytometric analysis of renal granulocyte infiltration. (**A**) Gating strategy: Pre–gating on live cells using Fixable Viability Dye eFluor 660 and further gating on single cells. (**B**) Representative flow cytometry data of infiltrating Ly6G–positive cells (granulocytes) and F4/80–positive cells (macrophages) in sham and I/R–injured kidneys of WT and *Trpv4* KO mice. (**C**) Quantification of infiltrating Ly6G–positive cells and (**D**) F4/80-positive cells. n = 4 for sham-operated WT and *Trpv4* KO mice and n = 6 for I/R–injured WT and *Trpv4* KO mice, two-way ANOVA, Sidak’s multiple comparisons test. In both cases P_Treatment_ < 0.0001, and P_Genotype_ and P_Interaction_ are not significant.

Renal IRI is associated with infiltration of granulocytes and monocytes/macrophages. These cells contribute to inflammation and subsequent repair of the injured kidney. Therefore, we characterized renal granulocyte and macrophage infiltration after IRI by flow cytometry in WT and *Trpv4* KO. Whole kidney cell suspensions were immunolabeled for Ly6G and F4/80 as markers for granulocytes and macrophages, respectively. Among pre–gated singlet live cells (Fig. 5A), Ly6G–positive & F4/80–negative granulocytes as well as Ly6G–negative & F4/80–positive macrophages were detected (Fig. 5B). No significant differences in granulocyte or macrophage populations were detected between IRI–damaged kidneys of WT and *Trpv4* KO mice at 24 hours after IRI (Fig. 5C,D). Renal cell populations were also similar in kidneys of *Trpv4* KO and WT mice which underwent sham surgery (Fig. 5C,D). We also performed immunohistochemistry to detect IRI-induced granulocyte infiltration, and counted the average number of Ly6B-positive cells in the outer medulla after IRI. Similarly to our flow cytometry results, no differences in granulocyte counts were detected between WT and *Trpv4* KO mice (Supplemental Fig. 6A,B).

### The Impact of TRPV4 Deficiency on Primary PTCs Treated with Cobalt Chloride (CoCl_2_)

**Figure 6:**
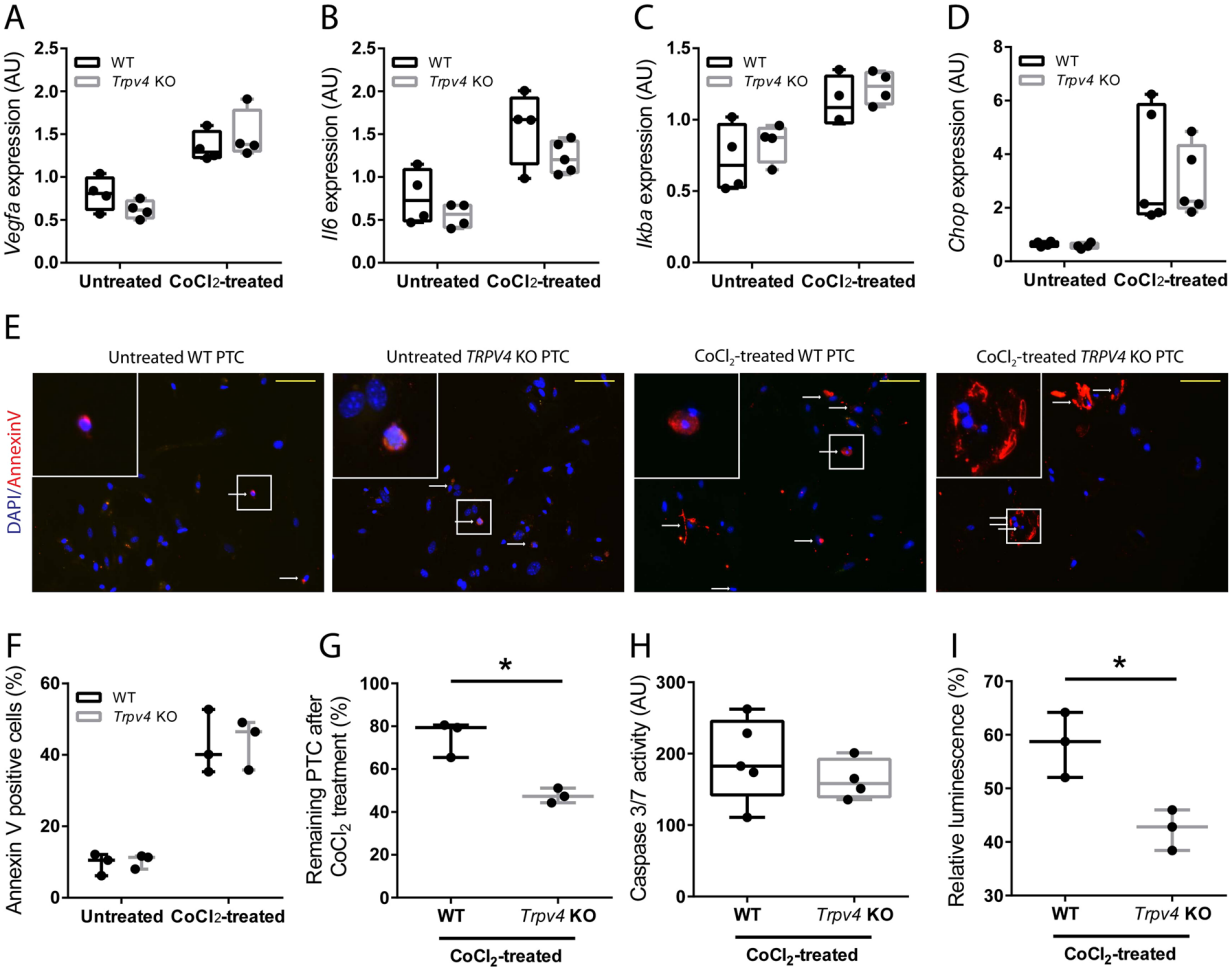
Treatment of primary proximal tubular cells (PTCs) of WT and Trpv4 KO mice with hypoxia-inducible factor 1-alpha (HIF1α)-stabilizing agent CoCl_2_. mRNA expression of (**A**) HIF1α target gene Vegfa, (**B**) stress cytokine interleukin 6 (Il6), bona fide NF-κB target gene nuclear factor of kappa light polypeptide gene enhancer in B-cells inhibitor alpha (IkBa), and (**D**) endoplasmatic reticulum stress marker CCAAT/enhancer binding protein homologous protein (Chop). Each dot is a mean of 2 technical repeats and represents a pool of PTCs isolated from 2–3 animals of the same genotype. Data of two independent experiments. n = 4 for untreated and n = 5 for CoCl_2_-treated WT and Trpv4 KO PTCs, two-way ANOVA. In all cases P_Treatment_ < 0.05, and P_Genotype_ and P_Interaction_ are not significant. (**E**) Representative ×200 images of Annexin V-stained (red) untreated WT and Trpv4 KO PTCs and of PTCs 24 hours after treatment of 300 μM hypoxia-mimetic agent CoCl_2_. Nucleus was stained with 4’,6-diamidino-2-phenylindole (DAPI) (blue). White arrows indicate Annexin V–positive tubular cells. Yellow bars represent 100 μm. (**F**) Quantification of Annexin V-positive PTCs in the respective groups. Each dot is a percentage of the mean Annexin V-positive cells/negative cells in 7–10 randomly selected high-power fields. Data of three independent experiments. Two-way ANOVA, P_Treatment_ < 0.05, and P_Genotype_ and P_Interaction_ are not significant. (**G**) Quantification of DAPI-positive WT and Trpv4 KO PTCs after a 24-hour treatment with 300 μM CoCl_2_. Each dot represents the percentage of the remaining DAPI-positive PTCs. To calculate the mean DAPI-positive PTC number of untreated and treated PTCs, 7–10 randomly selected high-power fields were counted and percentage was calculated. Data of three independent experiments. (**H**) Caspase 3/7 activity measured by the Caspase-Glo 3/7 Assay. Each dot is a ratio of the relative fluorescence of CoCl_2_-treated PTC/untreated PTC (background). n = 5 for CoCl_2_-treated WT and Trpv4 KO PTCs. (**I**) Percentage of viable PTCs measured by CellTiter-Glo Luminescent Cell Viability Assay. Luminescence of WT and Trpv4 KO PTCs after a 24-hour treatment with 300 μM CoCl_2_ was measured and was divided by the luminescence of their respective untreated controls and multiplied by 100. Data of three independent experiments, each experiment is a mean of 3–5 samples.

The stabilization of hypoxia-inducible factor (HIF)1α protein is a hallmark of hypoxia. To mimic hypoxia *in vitro*, WT and *Trpv4* KO PTCs were exposed to the HIF1α stabilizing agent CoCl_2_ in a low glucose medium for 24 hours. mRNA expression of the HIF1α target gene vascular endothelial growth factor (*Vegfa*) was upregulated by CoCl_2_ treatment in both WT and *Trpv4* KO PTCs, indicating successful HIF1α-stabilization. We next measured mRNA levels of the stress cytokine interleukin 6 (*Il6*), of nuclear factor of kappa light polypeptide gene enhancer in B-cells inhibitor, alpha (*IkBa*), a bona fide NF-κB target gene, and of the endoplasmic reticulum stress marker CCAAT/enhancer-binding protein homologous protein (*Chop*). In both WT and *Trpv4* KO PTCs CoCl_2_ treatment caused a similar upregulation of these stress genes (Fig. 6A–D). We subsequently counted the average number of cells per view-field of untreated and CoCl_2_-treated WT and *Trpv4* KO PTCs by microscopic analysis. Percentage of remaining PTCs were calculated and percentage of apoptotic PTCs were determined using Annexin V staining (Fig. 6E–G and Supplemental Fig. 8). To corroborate these data caspase 3/7 activity was determined using the Caspase-Glo 3/7 Assay (Fig. 6H) and number of viable cells were determined by the CellTiter-Glo Assay (Fig. 6I). In agreement with our *in vivo* data, we did not find any differences in the rate of apoptosis between WT and *Trpv4* PTCs, whereas fewer *Trpv4* PTCs could be detected after CoCl_2_ treatment when compared to WT PTCs suggesting an increased rate of necrotic cell death in *Trpv4* KO PTCs. Low-glucose environment by itself - without CoCl_2_ - did not lead to increased cell death in *Trpv4* PTCs compare to WT PTCs (Supplemental Fig. 9).

## Discussion

Acute kidney injury is a global public health problem in developed and developing countries. Recent data show that AKI is responsible for more than 1.5 million deaths yearly worldwide^36^. Thus, there is an urgent need for new therapeutic approaches. Recent pre–clinical data indicate that TRPV1 agonists can improve the outcome of ischaemic AKI^37,38^. Besides TRPV1, TRPV4 has been recently identified to serve as a major calcium influx pathway in endothelial cells to control vascular function^26,39^. Very recently, we were able to show that TRPV4 channels can promote endothelium-dependent relaxation in different vessels in the kidney, including vasa recta^25^. Others have demonstrated that TRPV4 plays an important role in the preservation of vasodilatation under hypoxic conditions^27^. Moreover, TRPV4 channels are highly expressed in renal tubular cells as shown in this study and by others^40^. We therefore hypothesized that loss-of-function of the osmotically-sensitive ion channel TRPV4, would impact the ability of tubular cells to recover from IRI.

In this study, we explored the pathophysiological role of TRPV4 in renal IRI. We uncovered massive necrosis of PTCs after renal IRI and provide evidence – contrasting to earlier reports – that PTCs express functional TRPV4. As early as 6 hours after renal IRI *Trpv4* KO mice showed higher serum creatinine levels compared to WT mice, which was maintained over 24 hours after renal ischaemia. Surprisingly, renal expression of the AKI markers *Ngal* and *Kim1*, which are expressed by distal and proximal tubular cells, respectively, did not differ between WT and *Trpv4* KO mice. In the kidney, NGAL and KIM1 have been reported to be expressed specifically in injured nephrons^19,41^. On the other hand, the expression profile of NGAL and KIM1 in the injured kidney has been described to be similar to TRPV4 expression in the intact kidney^18–20,41^. Therefore, one possible explanation for the lack of an increased *Ngal* and *Kim1* expression Trpv4 KO mice after renal IRI could thus be the higher necrosis rate of TRPV4 deficient tubular cells, which will in parallel deplete the number of NGAL and KIM1 expressing cells making the evaluation of renal *Ngal* and *Kim1* expression ambiguous in this study. Noteworthy, histological analysis of the IRI–damaged kidneys of *Trpv4* KO mice revealed massively increased cortical tubular damage, in particular tubular necrosis and loss of aquaporin-1 positive proximal tubular cells, compared to the kidneys of WT mice which underwent the same surgical procedure. This finding surprised us for two reasons; first, proximal tubular cells were thus far unknown to express TRPV4 channels, and second, the renal cortex is known to be less prone to ischemic injury as compared to the outer stripe of the outer medulla^18,20,41^. Therefore, we performed diligent co-immunostainings which revealed that proximal tubular cells indeed highly express TRPV4. While our study was underway Janas and co-workers reported on the expression of TRPV4 in the mouse kidney by using qPCR on isolated nephron segments, immunohistochemistry, and a *Trpv4* reporter mouse model^40^. Similarly to our findings they found TRPV4 to be expressed in the proximal tubule, in the distal convoluted tubule, and in the collecting duct, but not in the glomerulus and the thick ascending limb. These and our findings together revise earlier reports which could not demonstrate expression of TRPV4 in proximal tubular cells.

Tissue injury resulting from hypoxia can result in both necrosis and apoptosis. Apoptosis occurs in normal and disease states, whereas necrosis is induced only when cells or tissues are exposed to severe and acute injury^42^. Lack of TRPV4 has recently been linked to increased apoptosis and inhibited autophagy in a rat liver stellate cell line^43^. Therefore, we addressed the question whether a higher rate of apoptosis might be responsible for the extensive renal tubular damage in *Trpv4* KO mice. Interestingly, injured kidneys of *Trpv4* KO mice showed similar numbers of TUNEL–positive tubular cells as injured kidneys of WT mice. Moreover, *in vitro* treatment of WT and *Trpv4* KO PTCs with the HIF1α-stabilizing agent CoCl_2_ caused similar levels of apoptosis as measured by either the caspase 3/7 assay or by counting the number of Annexin V positive PTCs. In addition, mRNA levels of stress genes like *Il6*, *IkBa* and *Chop* were similarly upregulated in CoCl_2_-treated WT and *Trpv4* KO PTCs. However, CoCl_2_ treatment induced more cell death in *Trpv4* KO PTCs compared to WT PTCs as assessed by direct counting and by luminescent cell viability assay. These data suggest that TRPV4 deficiency induces necrosis but does not interfere with cell apoptosis in a hypoxia model of HIF1α stabilization. To the best of our knowledge no HIF binding sites on the *Trpv4* gene has been established. Nevertheless, it has recently been shown that HIF1α transcriptionally regulates *Trpa1* expression presumably via binding to an unusual hypoxia-response element-like motif within the *Trpa1* gene, which subsequently leads to suppressed secretion of interleukin-6 and interleukin-8^44^. One might speculate that a similar mechanism could regulate *Trpv4* expression in response to hypoxia. This in turn might be an indispensable mechanism for successful adaptation of PTCs to hypoxia.

Renal tubular epithelial apoptosis can initiate reperfusion–induced inflammation which is a hallmark of renal IRI^35,45^. Similar to the results for TUNEL–positive renal tubular cells, we found comparable degrees of infiltrating neutrophil granulocytes and F4/80–positive macrophages as well as mRNA expression levels of pro-inflammatory cytokines in IRI–kidneys of WT and *Trpv4* KO mice. These finding in combination with our histological and immunofluorescence results strongly suggests that the lack of functional TRPV4 does not increase inflammation and apoptosis, but directly compromises the ability of the renal epithelium to recover from ischaemic injury leading to prompt necrosis. Higher serum LDH levels in *Trpv4* KO mice compared to WT mice after renal IRI support this concept.

Calcium entry is a hallmark of the cellular reaction to ischemia in numerous cell types, including tubular epithelial cells. TRPV4 channels have been shown to be involved in ischemia-induced calcium entry in a number of cells, including reactive astrocytes and cardiac myocytes^46,47^. One may therefore expect that lack of TRPV4 channels should be rather beneficial. On the other hand, pharmacological inhibitors of TRPV4 channels are able to reduce cytokine production and preserve endothelial cell function, including Ca^2+^ signaling, and endothelium-dependent vasodilation, resulting in increased survival e.g. in septic mice^48^. Additionally, *Trpv4* KO mice exhibit higher blood sodium concentrations and present impaired osmotic sensitivity upon challenge^17^. Sublethal tubular damage results in impaired proximal sodium reabsorption which is expected to activate the tubular glomerular feedback mechanism leading to increased afferent vascular resistance and a concomitant decrease in glomerular filtration rate^49^. The impaired tubular transport of sodium in *Trpv4* KO mice might also contribute to the progression of IRI in a tubular glomerular feedback-dependent manner. In a recent study, the exogenous TPRV4 activator apigenin reduced hypertension-induced renal fibrosis through the AMP-activated protein kinase/sirtuin 1 pathway in a deoxycorticosterone acetate (DOCA)–salt rat model. Knockout of TRPV4 in mice abolished the beneficial effects of apigenin that were observed in the DOCA–salt hypertensive rats, suggesting a protective and anti-fibrotic role of TRPV4 in chronic renal disease^50^. Here, our data suggest that lack of TRPV4 impairs recovery after acute renal IRI and induces massive-scale necrosis, especially in the renal cortex.

We demonstrated earlier that activation of TRPV4 promotes endothelial relaxation in renal resistance arteries, renal conduit arteries and in the medullary vasa recta^25^. During renal IRI the most commonly injured epithelial cells are the proximal tubular cells. These cells are particularly susceptible to ischemic insult partly due to their high metabolic rate required for mediating ion transport and a limited capacity to undergo anaerobic glycolysis^51^. As *Trpv4* KO mice exhibited significantly more necrosis of proximal tubular cells after renal IRI compared to WT mice, it is tempting to speculate that vascular mechanisms may also be responsible for the observed effect.

A limitation of our study is that we used *Trpv4* pan-null mice. Global lack of TRPV4 can lead to a number of pathologies e.g. impaired osmotic and mechanosensing^17^ which become more prominent in stress situations. Although we carefully designed our study to circumvent possible confounding effects caused by the baseline phenotype of the *Trpv4* KO mice, we cannot exclude that the above-mentioned limitation of global TRPV4 deficiency could have influenced the outcome of our study. This might apply particularly for haemodynamic changes after renal ischemia, since TRPV4 has an established role in the maintenance of vascular tone^39^. In addition, the increased sensitivity to ischaemia may not only be due directly to TRPV4 loss-of-function, but due to developmental-compensatory and other secondary mechanisms caused by genetically-engineered global absence on Trpv4. Indeed, TRPV4 have been recently described as a sensor of cell stress in the diseased fatty liver. These data lend solid support to the emerging concept of TRPV4 protecting a key epithelial cell – hepatocytes – from oxidative injury^52^.

In summary, genetically-encoded Trpv4 knockout (pan-null) aggravates acute renal tubular injury which is associated with enhanced renal epithelial necrosis. Although the results of our study suggest that this necrosis may be a direct effect of TRPV4 deficiency, additional studies are needed to clarify the exact molecular mechanism. Further studies using cell–specific TRPV4 mouse models are needed to clarify the precise pathophysiological sequence of events, and to clarify how the IRI–induced, TRPV4–dependent tubular damage influences long term renal recovery and survival.

## Methods

### Mice

*Trpv4* KO mice have been generated and characterized previously.^17^ Wild–type (WT) control mice as littermates on the same genetic background were used. Mice were allowed free access to standard chow and water and were kept in a 12:12–h light–dark cycle. Animal studies were approved by the Berlin Animal Review Board in 2012 (No. G0082/12) and conducted in accordance with the standards of the American Physiological Society.

### Renal IRI Model

Renal IRI was induced as described earlier^53^. Briefly, male mice (aged 14–17 weeks) were anaesthetized by isoflurane (2.3%) in air (350 mL/min). Mice were operated individually to ensure similar exposure to isoflurane (33.8 ± 3.0 min, mean ± SD)^54^. Body temperature was maintained at 37 °C and monitored during surgery using a temperature controller with a heating pad (TCAT–2, Physitemp Instruments). Ischaemia was induced after right–sided uni-nephrectomy by clipping the pedicle of the left kidney for 20 minutes with non–traumatic aneurysm clips (FE690K, Aesculap). Reperfusion was confirmed visually and the abdomen and the skin were sutured separately with a 5/0 braided-silk suture. After surgery mice had free access to water and chow. Body–warm sterile physiological saline solution (1 mL) and pre–emptive analgesia with tramadol (1 mg/kg) were applied subcutaneously to every mouse. Sham operation was performed in a similar manner, except for clamping the renal pedicle. Mice with bleeding during surgery, with incomplete renal reperfusion, with excessive exposure to isoflurane for any reason, with significant temperature fluctuation during surgery, or with signs of infection 24 h after IRI, were immediately euthanized and not used for further analysis. Twenty–four hours after reperfusion, mice were sacrificed, and kidney and blood samples were collected for further analysis. The kidneys were divided into two portions. Half of the kidney was immersed in 4% phosphate–buffered saline (PBS)–buffered formalin for histology, and the other half was snap–frozen in liquid nitrogen for RNA preparation.

### Primary Proximal Tubular Cell Isolation and Calcium concentration (Ca^2+^) measurement

Cells were obtained and cultured as described previously^53^. Briefly, WT and *Trpv4* KO mice aged 10–12 weeks were euthanized by an overdose of isoflurane and the kidneys were flushed with 10 ml ice-cold PBS (Sigma). Renal cortices were dissected visually and minced into small pieces which were transferred through two layers of stainless steel sieves (pore size 125 μm and 106 μm; Linker). Tubular fragments caught by the 106 μm sieve were flushed in the reverse direction with PBS and centrifuged for 5 minutes at 170 × g, washed, and then resuspended into the appropriate amount of culture medium: 1:1 DMEM/F12 (D 8437; Sigma) supplemented with fetal calf serum (FCS) 10%, penicillin 100 IU/ml and streptomycin 100 µg/ml buffered to pH 7.4. Plates were incubated in a standard humidified incubator at 5% CO_2_. Medium was changed two days later and maintained every other day until the monolayer of cells reached 90% confluence; at this time, over 90% of the isolated cells were megalin-positive (sheep-anti-LRP2 antibody (1:10000) was a gift from Prof. Thomas Willnow (Max–Delbrück Center for Molecular Medicine, Berlin); secondary antibody was donkey anti-sheep Alexa 555 (1:2000; Thermo Fisher Scientific)).

After 5–7 days cells were trypsinized and seeded on a 48-well plate at a density of 20,000 cells/well. Cells were let to adhere overnight. The next day cells were loaded with the Ca^**2+**^ indicator Fluo-4 AM (Invitrogen, F14201) at a final concentration of 10 µM at 37 °C in a standard humidified incubator at 5% CO_2_ in the presence of Pluronic F127 (0.005%; w/v, Calbiochem, 540025). After 1-hour incubation time, cells were washed twice with pre-warmed PBS and then stimulated with the TRPV4 agonist GSK1016790A (Sigma-Aldrich) at final concentrations of 10 and 100 nM. Unstimulated cells of each genotype served to measure background fluorescence. After stimulation fluorescence was measured in a SpectraMax M5 Multi-Mode Microplate Reader (Ex.: 488 nm, Em.: 535 nm) using the SoftMax Pro software package. Data are expressed as (F − Fo)/Fo, where F is the fluorescence intensity of GSK1016790A-treated cells and Fo is the fluorescence intensity of the unstimulated cells.

### Quantitative Real–Time (qRT)–PCR

qRT–PCR was performed as described earlier^53^. Briefly, total RNA was isolated from snap–frozen kidneys after homogenization with a Precellys 24 homogenizer (Peqlab) using RNeasy RNA isolation kit (Qiagen). RNA quality and concentration were determined by a NanoDrop–1000 spectrophotometer (Thermo Fisher Scientific). Two micrograms of total renal RNA were transcribed to cDNA (Applied Biosystems). Quantitative analysis of target mRNA expression was calculated using the relative standard curve method. TaqMan and SYBR green analysis was conducted using an Applied Biosystems 7500 Sequence Detector (Applied Biosystems). The expression levels were normalized to the purinergic housekeeping gene hypoxanthine-guanine phosphoribosyltransferase. Primer sequences are provided in Supplementary Table 2.

### Serum Measurements

To allow for repeated measurement of lab parameters within a short time interval in the same mouse, 90 μL blood was taken from the facial vein and parameters were measured using an i-STAT system with Chem8+ cartridges (Abbott). Serum creatinine levels of sham-operated mice were measured by Labor 28 GmbH (Berlin, Germany). Lactate dehydrogenase was measured at the Pathophysiological Platform of the Max–Delbrück Center for Molecular Medicine (Berlin, Germany) using a Fuji DRI-CHEM automated clinical chemistry analyser.

### Microscopy

#### Histology

Formalin–fixed, paraffin–embedded, 5 µm thick kidney sections were stained with haematoxylin (Sigma) and 1% eosin (Sigma). Semi-quantitative scoring of tubular damage was performed in a blinded manner in that 10 to 18 images at 200× magnification per sample were scored on a 1 to 10 scale according to percentage (where 0 = 0% and 10 = 100%) of tubular dilation, brush border loss, tubular necrosis, tubule degeneration and tubular cast formation. The total score was calculated as sum of all five morphological parameters.

#### Immunofluorescence

Five μm thick cryosections were post-fixed in ice-cold acetone, air-dried, rehydrated and blocked with 10% normal donkey serum (Jackson ImmunoResearch) for 30 min. Next, sections were incubated overnight at 4 °C with the following primary antibodies: rat anti-Ly-6B.2 (Gr1) (1:300; MCA771G; AbD Serotec), rabbit anti-TRPV4 (1:50; acc-034, Alomond Labs), rabbit anti-aquaporin-1 (1:300; AB2219, Millipore), rabbit anti-sodium-chloride symporter (1:500; AB3553; Millipore), goat anti-aquaporin-2 (1:200, sc-9882; Santa Cruz Biotechnologies), rabbit anti-sodium-potassium-chloride cotransporter 2 (1:1000; a gift from Sebastian Bachmann, Charité Berlin). For co-localization experiments serial sections were stained. All incubations were performed in a humid chamber. For fluorescence visualization of bound primary antibodies, sections were further incubated with appropriate Cy3- or Alexa 488-conjugated secondary antibodies (1:500; Jackson ImmunoResearch) for 1 h in a humid chamber at room temperature.

CoCl_2_ and untreated primary proximal tubular cells isolated from WT and Trpv4 KO mice (see below) were stained with Annexin V (51-65875×; BD Pharmingen) and DAPI (final concentration 1 µg/mL). Annexin V positive cells and negative cells were counted in 7–10 randomly selected high-power fields (400×) using of a Zeiss Axio Imager.M2 fluorescence upright microscope.

### TUNEL Assay

TMR *In Situ* Cell Death Detection Kit (Roche) was used according to the manufacturer’s instructions on formalin–fixed, paraffin–embedded 2 μm thick sections. In order to visualize cell nuclei slides were counterstained with the nuclear dye 4′,6-diamidino-2-phenylindole (DAPI, Sigma). The number of TUNEL–positive tubular cells was determined as mean of at least 5 non–overlapping fields in the cortex by use of a Zeiss Axio Imager.M2 fluorescence upright microscope using a 20× Plan–Apochromat objective.

### Flow Cytometry

Granulocyte and macrophage infiltration was assessed in sham–operated and IRI–injured kidneys. GentleMacs C-tubes (Miltenyi Biotec) in the presence of 10 mg/mL collagenase IV (Sigma) and 200 U/mL DNase I (Roche) dissolved in Hank’s balanced salt solution were used to obtain single cell suspensions. Fixable Viability Dye eFluor 660 (eBioscience) was used for detection of dead cells. Granulocytes and macrophages were stained with phycoerythrin (PE)–conjugated anti–Ly6G (clone: 1A8, Beckton Dickinson) and eFluor450–conjugated anti–F4/80 (clone: BM8, eBioscience) antibodies, respectively. Samples were analyzed on a FACSCanto II flow cytometer (Becton Dickinson). Data analysis was conducted by FlowJo (TreeStar) software.

### HIF1α Stabilization in Primary PTC

Primary proximal tubular cells isolated from WT and *Trpv4* KO mice were treated with 300 μM CoCl_2_ to stabilize HIF1α or left untreated in a low-glucose DMEM medium (D6046, Sigma) for 24 hours. Low glucose medium was used because it is known that cell death in proximal tubular cell cultures is HIF1α-dependent only when glucose availability is limited^55^. After 24 hours medium was removed and cells were harvested for further analysis. To count Annexin V and DAPI-positive WT and Trpv4 KO PTC, cells were seeded at a density of 10^4^ cells/well in a chamber slide with a removable 8 well silicone chamber (Ibidi). Untreated PTC and PTC treated with 300 μM CoCl_2_ for 24 hours were stained with Annexin V (100 µL binding buffer +5 µL Annexin V/well) after washing with PBS. After 15 minutes incubation in the dark at room temperature the staining solution was removed and PTC were fixed with pre-warmed (37 °C) 4% formaldehyde (200 µL/well) for 30 minutes in the dark at room temperature. Thereafter the PTC were washed, the chamber was removed and the slide was subjected to microscopic analysis.

### Caspase-Glo 3/7 and CellTiter-Glo Luminescent Cell Viability Assays

96-well clear plates were seeded with 1 × 10^4^ WT or *Trpv4* KO PTC/well. Cells were allowed to attach overnight. On average, cells were at 50% confluency before addition of 300 μM CoCl_2_. Twenty-four hours after CoCl_2_ treatment the cells were assayed by the Caspase-Glo 3/7 or by the CellTiter-Glo Luminescent Cell Viability Kit according to manufacturer’s instructions (Promega). After 10 min on a plate shaker at room temperature, 90% of the lysate volume was transferred to a 96-well solid-white plate (Nunc). Cell lysates were analysed in a SpectraFluor Plus microplate reader (Tecan), and blank values were subtracted from data points. Relative fluorescence intensity of treated cells was normalized by their respective untreated control cells to normalize for possible seeding density differences between WT and *Trpv4* KO PTC. In case of the CellTiter-Glo Luminescent Cell Viability Assay an ATP standard curve was measured in every experiment.

### Statistics

Statistical analysis was performed using GraphPad Prism 5.04 (GraphPad Software). Normality of the data was evaluated by Kolmogorov–Smirnov test. Grubbs’ test was used to detect outliers. Data were analysed by repeated measurement or regular two-way ANOVA with Sidak’s multiple comparisons test, as appropriate. Data with two groups were tested by two-sided unpaired *t*–test (data with normal distribution). Data are presented as boxplots, showing median, 25 and 75 percentiles and minimum and maximum. Detailed tubular injury score data are presented as mean ± SD. P values < 0.05 were considered as statistically significant.

### Data availability

All data generated or analysed during this study are included in this published article (and its Supplementary Information files). Raw data is available to interested parties upon request.

## Electronic supplementary material

Supplementary Figures and Tables
