# Supplementary material for: Transient Receptor Potential Vanilloid 4 Channel Deficiency Aggravates Tubular Damage after Acute Renal Ischaemia Reperfusion
Source: Sci Rep. 2018 Mar 20;8:4878. doi: 10.1038/s41598-018-23165-0 (PMC5861116; doi:10.1038/s41598-018-23165-0)
Supplement: Supplementary file 1 — Supplementary Figures and Tables [file 41598_2018_23165_MOESM1_ESM.doc]

**Transient Receptor Potential Vanilloid 4 Channel Deficiency Aggravates Tubular Damage After Acute Renal Ischaemia Reperfusion**

**Short title:** TRPV4 in Acute Kidney Injury

Marwan Mannaa^1#^ MD, Lajos Markó^2#,*^ MD, PhD, András Balogh^2,3^ MD, PhD, Emilia Vigolo^4^, Gabriele N’diaye^2^, Mario Kaßmann^1^ PhD, Laura Michalick^5^, Ulrike Weichelt^5^ PhD, Kai M. Schmidt–Ott^4^ MD, PhD, Wolfgang B. Liedtke MD, PhD^6^, Yu Huang^7^, Dominik N. Müller^2,4^ PhD, Wolfgang M. Kuebler^5^ MD, Maik Gollasch^1,2,*^ MD, PhD

^1^Charité Campus Virchow, Nephrology/Intensive Care, Berlin, Germany

^2^Experimental and Clinical Research Center, a joint cooperation between the Charité Medical Faculty and the Max–Delbrück Center for Molecular Medicine, Berlin, Germany

^3^Department of Medical Biology, University of Pécs Medical School, Pécs, Hungary; Signal Transduction Research Group, János Szentágothai Research Centre, Pécs H-7624, Hungary

^4^Max–Delbrück Center for Molecular Medicine, Berlin, Germany

^5^Institute of Physiology, Charité Universitätsmedizin Berlin, Berlin, Germany

^6^Departments of Neurology, Neurobiology, and Clinics for Pain and Palliative Care, Duke University Medical Center, Durham, USA

^7^Institute of Vascular Medicine, Chinese University of Hong Kong, Hong Kong, China; Li Ka Shing Institute of Health Sciences, Chinese University of Hong Kong, Hong Kong, China

^#^These authors contributed equally to this work.

The authors have declared that no conflict of interest exists.

**Title length:** 15 words; **Word count of main text:** 3477; **Word count of the abstract:** 198

***Corresponding Authors:**

Dr. Lajos Markó or Dr. Maik Gollasch, Experimental and Clinical Research Center, Lindenberger Weg 80, 13125 Berlin, Germany, Tel: +49 30-450-540-177, Fax: +49 30-450-553-916, E-mail: lajos.marko@charite.de or maik.gollasch@charite.de

**Legends to Supplementary Figures**

**Figure 1.** Serum creatinine levels in sham-operated *Trpv4* KO and WT mice. n=4 for WT and *Trpv4* KO mice.

**Figure 2. Expression of proximal tubular cell marker aquaporin-1 in contralateral non-injured kidneys and after ischaemia-reperfusion injury in WT and *Trpv4* KO kidneys.** Representative cortical images of aquaporin-1 stain on sections of WT and *Trpv4* KO kidneys. White arrows point to aquaporin positive necrotic tubule. Yellow bar represents 100 μm.

**Figure 3. Fluorescence detection of Ca^2+^ influx in proximal tubular cells upon TRPV4 activation**. After loading isolated proximal tubular cells from WT and Trpv4 KO mice with the Ca^2+^ indicator Fluo-4 AM, the cells were stimulated with the TRPV4 agonist GSK1016790A at concentrations of 10 and 100 nM. Unstimulated cells of each genotype served to measure background fluorescence. Data are expressed as (F-Fo)/Fo, where F is the fluorescence intensity of GSK1016790A-treated cells and Fo is the fluorescence intensity of the unstimulated, Ca^2+^ indicator loaded cells. n=4-7/group, two-way ANOVA, Sidak's multiple comparisons test. *P<0.05.

**Figure 4. Renal injury the outer stripe of outer medulla.** (A) Representative outer stripe of outer medulla (OM) images of haematoxylin and eosin stained sections of sham and ischaemia/reperfusion (I/R)–injured kidneys of WT and Trpv4 KO mice (×200). (B) Semi–quantification of tubular injury in the outer stripe of OM. (C) Detailed analysis of tubular injury in the outer stripe of OM. Histological analysis were performed in n=5 for both WT and *Trpv4* KO mice, two-way ANOVA, Sidak's multiple comparisons test. *P<0.05. * on the histological images represent necrosis and ^#^ represent tubular cast formation.

**Figure 5.** **Detection of TRPV4 protein expression in the kidney by immunofluorescence.** Representative immunofluorescence images of WT and *Trpv4* KO mice sham kidneys. A strong staining is observed in WT kidneys, whereas no detectable signal is observed in the kidneys from *Trpv4* KO mice. Yellow bar represents 100 μm.

**Figure 6.** **Detection of tubular apoptosis on sections of contralateral kidneys of WT and *Trpv4* KO mice.** Representative ×200 images of TUNEL labelling on contralateral WT and *Trpv4* KO kidney sections. Yellow bars represent 100 μm.

**Figure 7.** **Immunofluorescent detection of Ly6B-positive cells (granulocytes) in I/R-injured kidneys of WT and *Trpv4* KO mice**. (A) Representative images (×200) and (B) quantification. n=5 for both WT and *Trpv4* KO mice.

**Figure 8.** **Treatment of WT and Trpv4 KO primary proximal tubular cells (PTCs) with hypoxia-inducible factor 1-alpha (HIF1α)-stabilizing agent** **CoCl_2_**. Representative ×200 images of Annexin V-stained (red) untreated WT and Trpv4 KO PTCs and of PTCs 24 hours after treatment of 300 μM hypoxia-mimetic agent CoCl2. Nucleus was stained with 4',6-diamidino-2-phenylindole (DAPI) (blue). Differential interference contrast was used to identify respective PTC. Yellow bars represent 100 μm.

**Figure 9. Treatment of WT and Trpv4 KO primary proximal tubular cells (PTCs) with low glucose containing medium.** Percentage of viable PTCs measured by CellTiter-Glo® Luminescent Cell Viability Assay. Luminescence of WT and Trpv4 KO PTCs after being in low-glucose medium was measured and was divided by the luminescence of their respective untreated controls and multiplied by 100. Data of three independent experiments, each experiment is a mean of 3-5 samples.

**Supplementary Figure 1.**

**
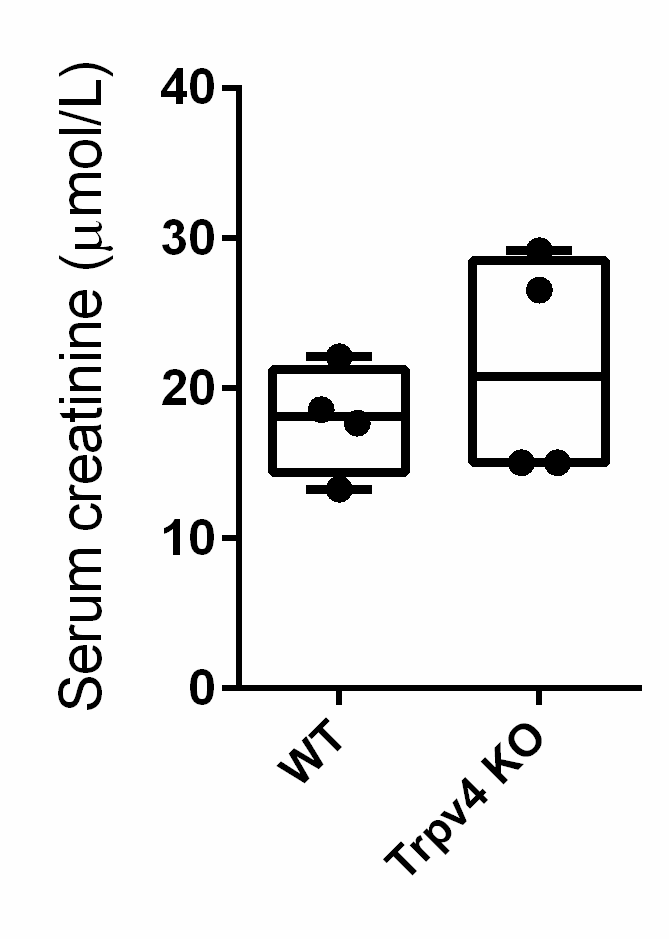
**

**Supplementary Figure 2.**

**
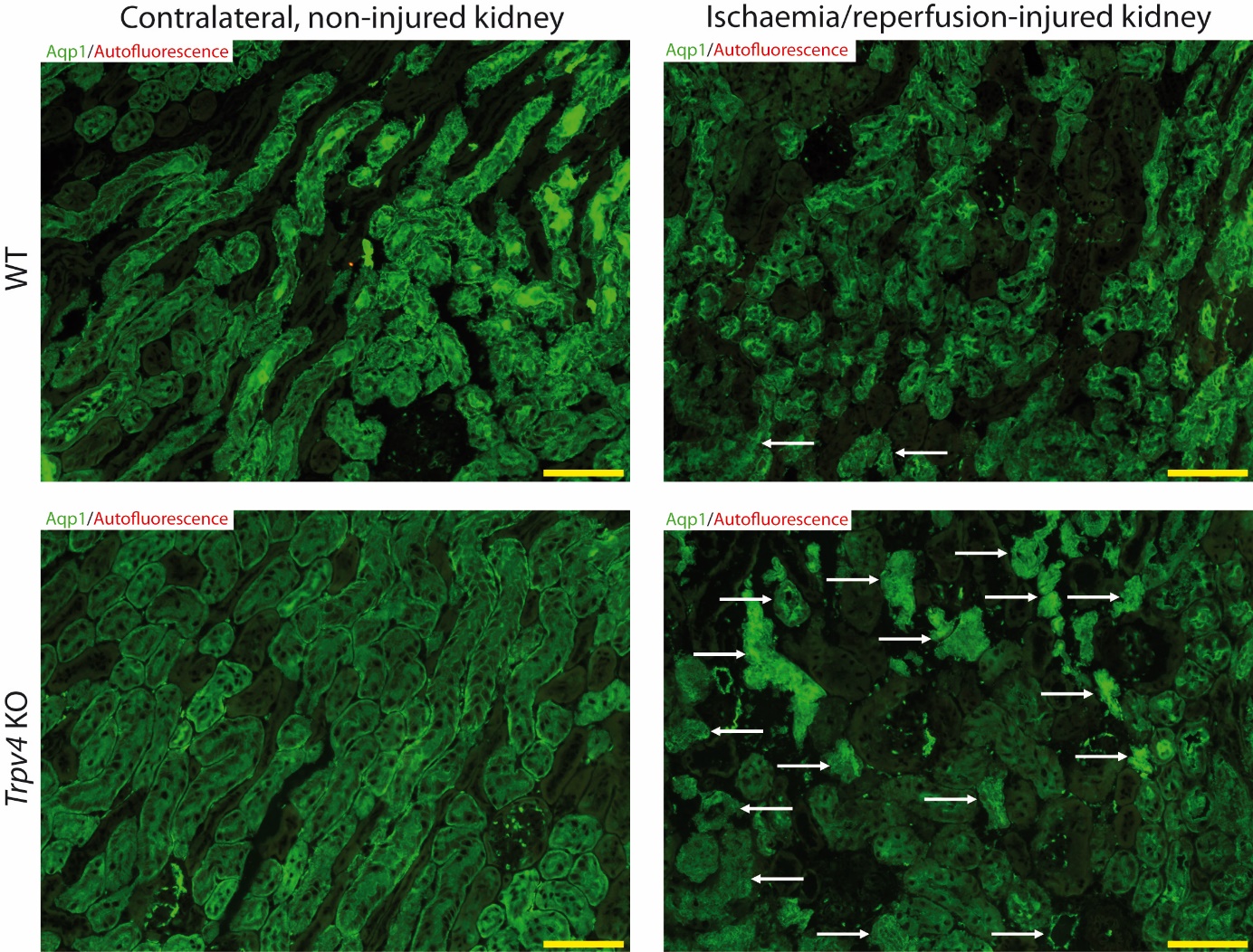
**

**Supplementary Figure 3.**


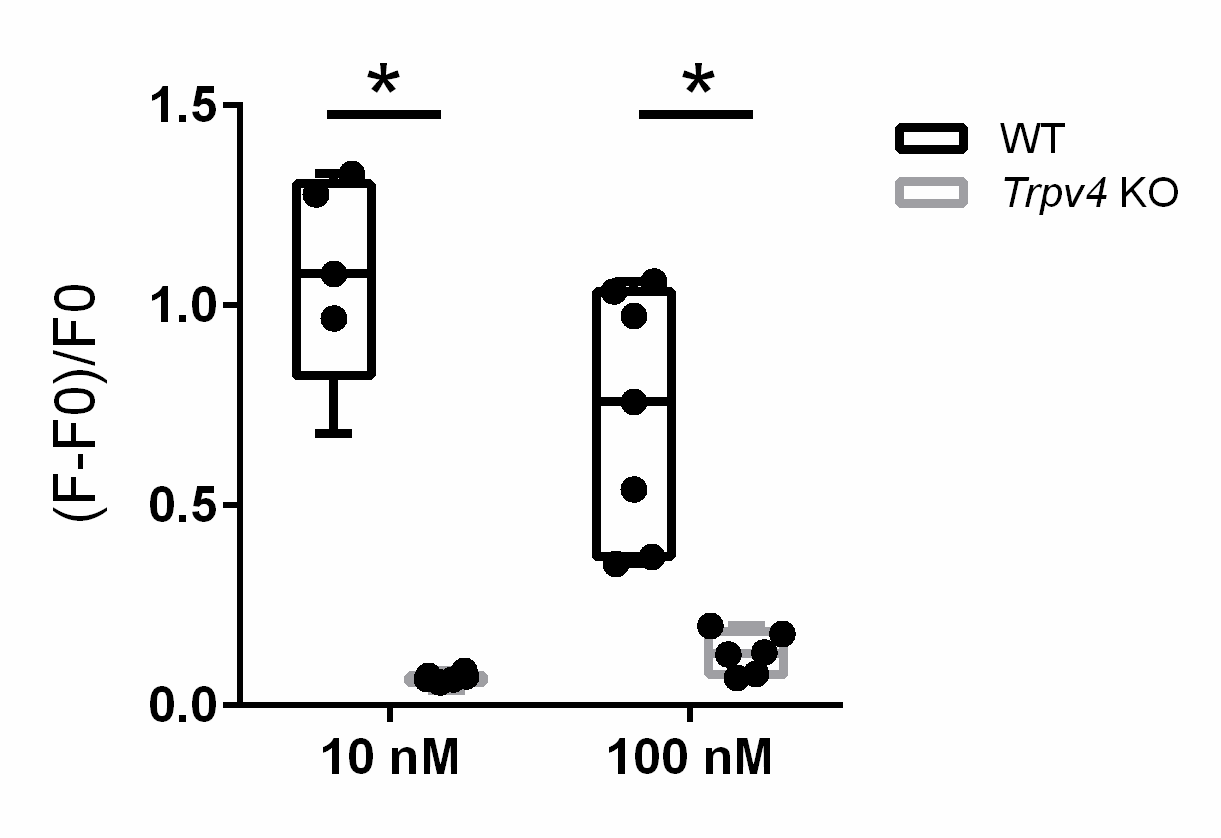


**Supplementary Figure 4**

**
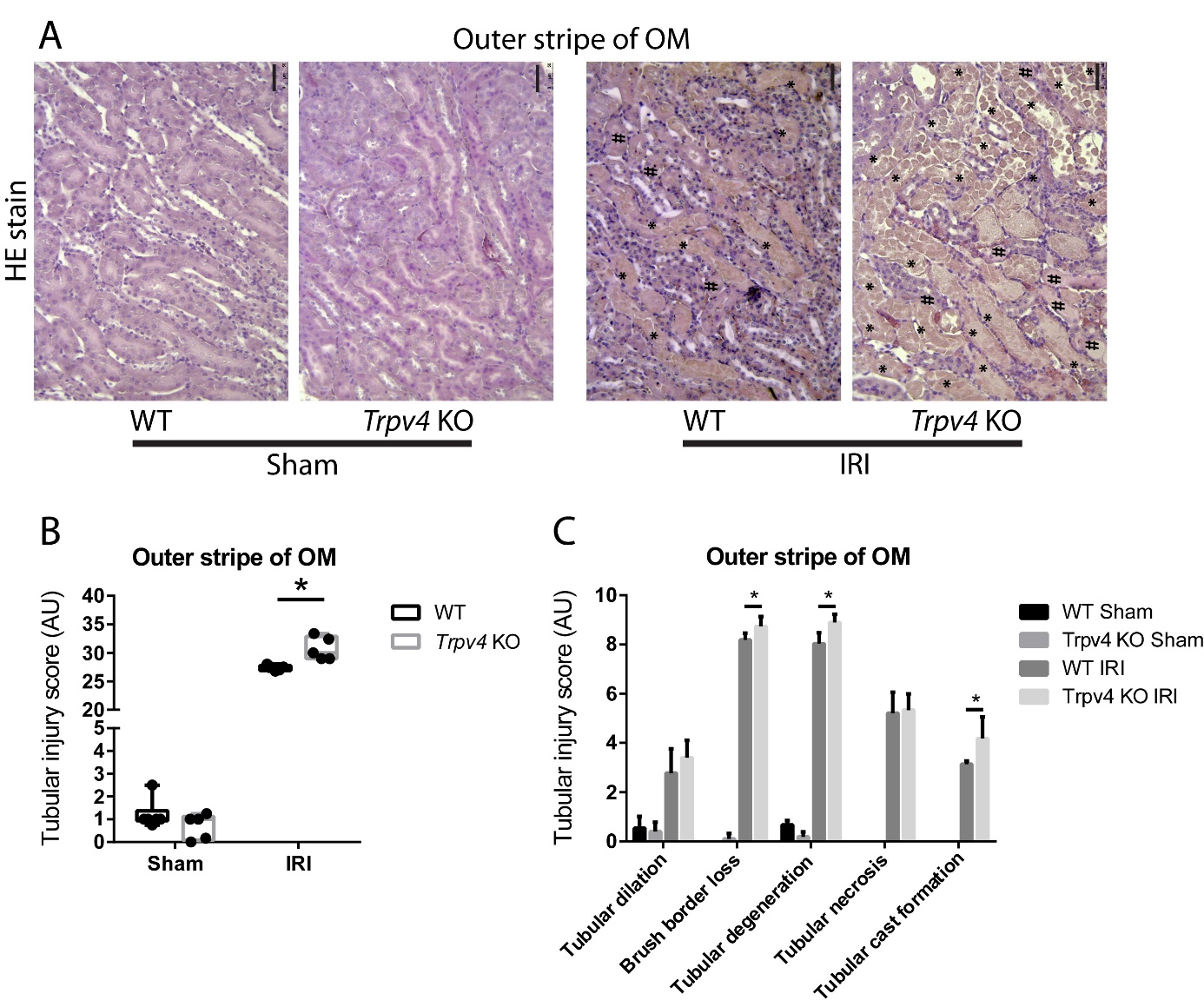
**

**Supplementary Figure 5**


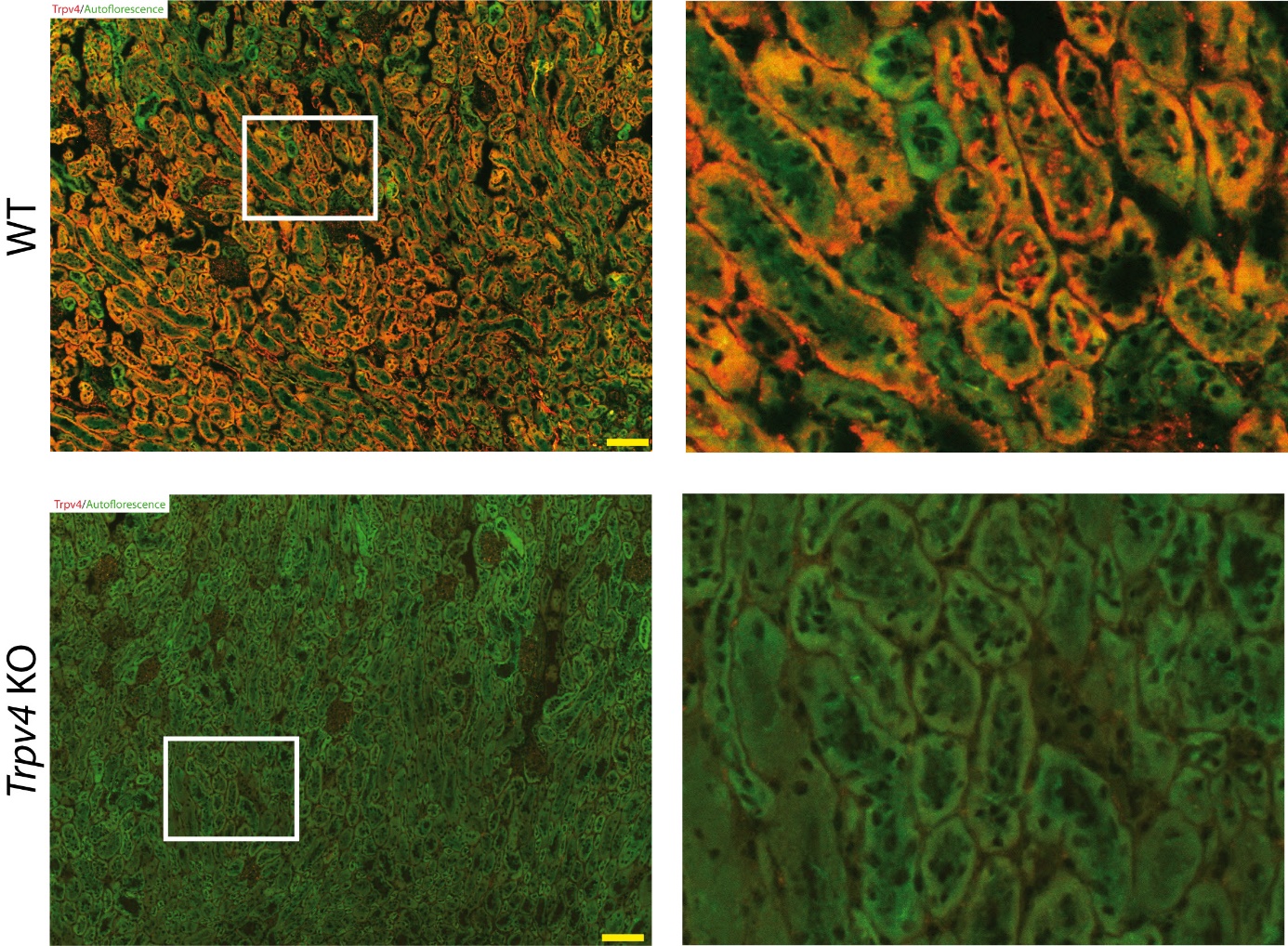


**Supplementary Figure 6**


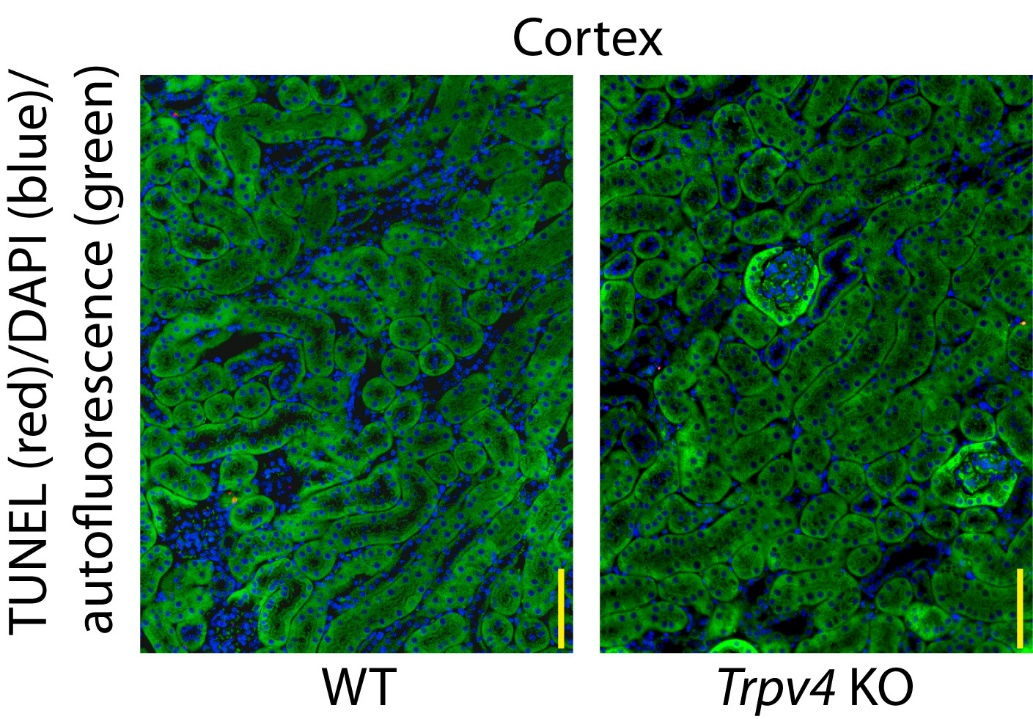


**Supplementary Figure 7**


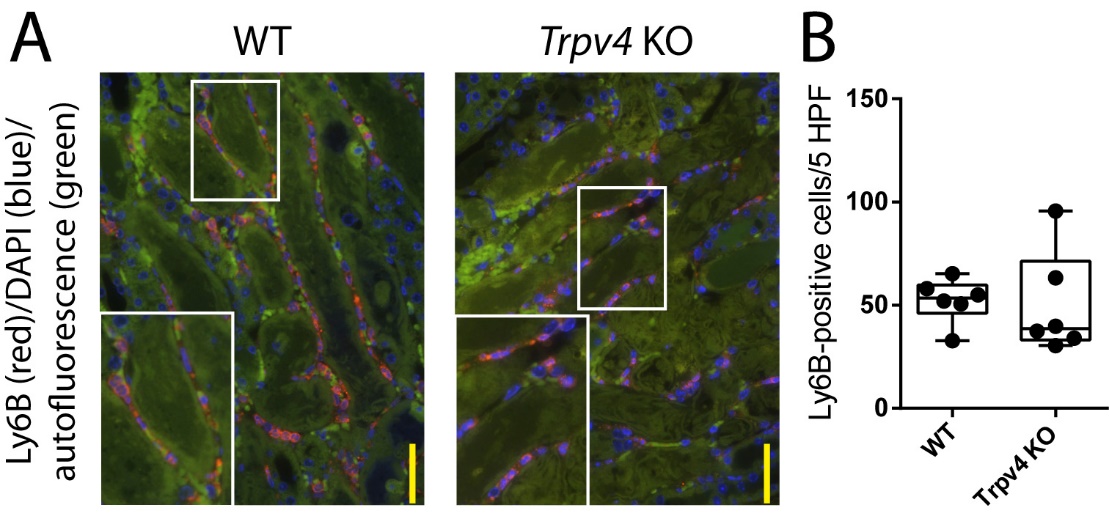


**Supplementary Figure 8**


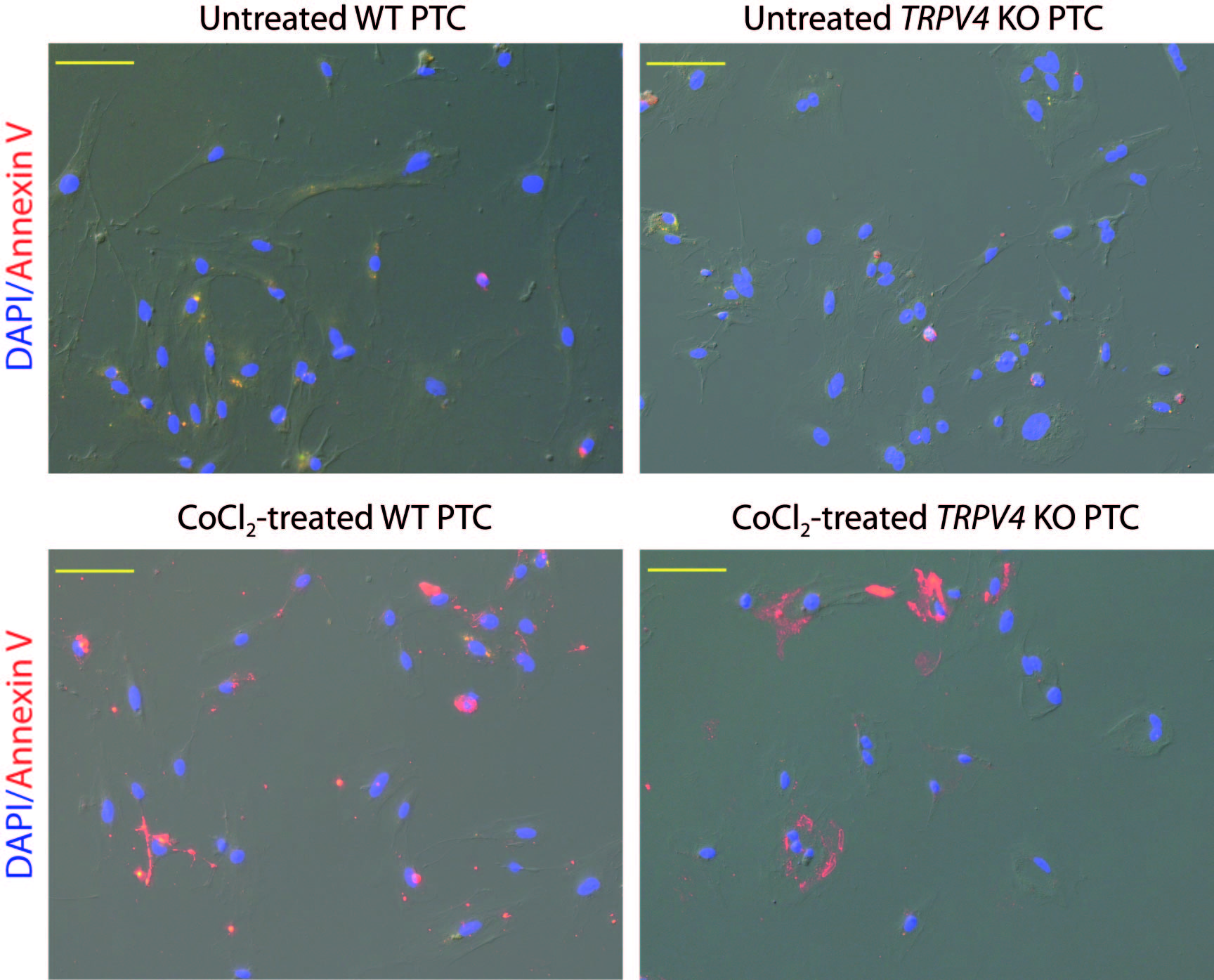


**Supplementary Figure 9**


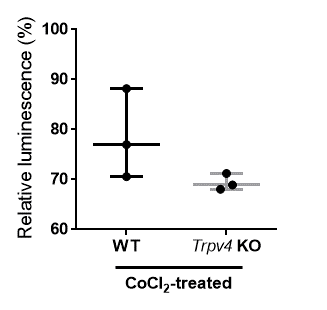


**Supplementary Table 1**. Serum parameters of WT and *Trpv4* KO control mice 6 hours and 24 hours after renal ischemia reperfusion injury (IRI). n.a.= not applicable.

| **6 h after IRI** |  | **WT** | | **Trpv4 KO** | |  |  |
| --- | --- | --- | --- | --- | --- | --- | --- |
| **Serum parameter** | **Unit** | **Mean** | **SD** | **Mean** | **SD** | **P-value** | |
| Sodium | mmol/L | 144,33 | 4,04 | 143,33 | 2,52 | 0,734 | |
| Potassium | mmol/L | 6,77 | 0,93 | 7,13 | 0,42 | 0,567 | |
| Chloride | mmol/L | 120,33 | 5,69 | 118,00 | 4,00 | 0,592 | |
| Ionized Calcium | mmol/L | 1,20 | 0,08 | 1,20 | 0,01 | 0,948 | |
| Total Carbon Dioxide | mmol/L | 15,00 | 1,73 | 14,33 | 2,08 | 0,692 | |
| Glucose | mg/dL | 107,00 | 13,89 | 110,67 | 15,37 | 0,775 | |
| Urea Nitrogen | mg/dL | 26,27 | 4,25 | 40,17 | 5,15 | 0,023 | |
| Hematocrit | % PCV | 36,00 | 4,24 | 38,67 | 5,86 | 0,625 | |
| Hemoglobin | g/dL | 12,25 | 1,48 | 13,13 | 1,97 | 0,632 | |
| Anion Gap | mmol/L | 17,00 | 2,00 | 19,00 | 1,00 | 0,196 | |

| **24 h after IRI** |  | **WT** | | **Trpv4 KO** | |  |
| --- | --- | --- | --- | --- | --- | --- |
| **Serum parameter** | **Unit** | **Mean** | **SD** | **Mean** | **SD** | **P-value** |
| Sodium | mmol/L | 146,33 | 9,02 | 141,67 | 2,08 | 0,432 |
| Potassium | mmol/L | 8,03 | 1,12 | 7,77 | 1,50 | 0,817 |
| Chloride | mmol/L | 120,00 | 10,44 | 113,67 | 4,04 | 0,383 |
| Ionized Calcium | mmol/L | 0,89 | 0,30 | 0,90 | 0,05 | 0,929 |
| Total Carbon Dioxide | mmol/L | 14,33 | 3,21 | 15,67 | 3,51 | 0,653 |
| Glucose | mg/dL | 74,00 | 11,53 | 104,00 | 21,70 | 0,102 |
| Urea Nitrogen | mg/dL | >50 | n.a. | >50 | n.a. | n.a. |
| Hematocrit | % PCV | 26,00 | 5,66 | 31,33 | 4,04 | 0,297 |
| Hemoglobin | g/dL | 8,85 | 1,91 | 10,67 | 1,33 | 0,288 |
| Anion Gap | mmol/L | 22,00 | 5,66 | 22,00 | 4,24 | 1,000 |

**Supplementary Table 2.** Primer sequences used in quantitative real-time PCR.

| Gene | Forward | Probe | Reverse |
| --- | --- | --- | --- |
| Hprt | 5'-GCTTTCCCTGGTTAAGCAGTACA-3' | - | 5'-ACACTTCGAGAGGTCCTTTTCAC-3' |
| Il1b | 5'-AGAAGAGCCCATCCTCTGTGACTCATGG-3' | 5'-FAM-AGAAGAGCCCATCCTCTGTGACTCATGG-TAMRA-3' | 5'-CACACACCAGCAGGTTATCATCA-3' |
| Havcr1 | 5'-CTGGAGTAATCACACTGAAGCAATC-3' | 5'-FAM- CTCCAGGGAAGCCGCAGAAAAACC-TAMRA-3' | 5'-GATGCCAACATAGAAGCCCTTAGT-3' |
| Il6 | 5'- TGTCTCGAGCCCACCAGG-3' | 5'-FAM-CGAAAGTCAACTCCATCTGCCCTTCAGG-TAMRA-3' | 5'- TGCGGAGAGAAACTTCATAGCTG-3' |
| Lcn2 | 5'-TGATCCCTGCCCCATCTCT-3' | 5'-FAM-TCACTGTCCCCCTGCAGCCAGA-TAMRA-3' | 5'-GGAACTGATCGCTCCGGAA-3' |
| Tnf | 5'-GGTCCCCAAAGGGATGAGAA-3' | 5'-FAM-TTCCCAAATGGCCTCCCTCTCATCA-TAMRA-3' | 5'-TGAGGGTCTGGGCCATAGAA-3' |
| Ikba | 5'-CTGCACACCCCAGCATCTC-3' | 5'-FAM-ACTCCGTCCTGCAGGCCACCAA-TAMRA-3' | 5'-CAGACACGTGTGGCCATTGT-3' |
| Vegfa | 5'-CTGTAACGATGAAGCCCTGGAG-3' |  | 5'-TGGTGAGGTTTGATCCGCAT-3' |
| Chop | 5'-CCACCACACCTGAAAGCAGAA-3' |  | 5'-AGGTGAAAGGCAGGGACTCA-3' |
